# Effects of resveratrol on postmenopausal women: a systematic review and meta-analysis

**DOI:** 10.3389/fphar.2025.1588284

**Published:** 2025-07-23

**Authors:** Weidong Wu, Tianwei Meng, Fangfang Jin, Junwei Li, Jiahao Huang, Zhuang Guo, Miao Yu, Yanyan Zhou

**Affiliations:** ^1^Key Laboratory of Basic Theory of Chinese Medicine, Heilongjiang University of Chinese Medicine, Harbin, China; ^2^Department of Internal Medicine, Heilongjiang University of Chinese Medicine, Harbin, China; ^3^ Graduate School, China Academy of Chinese Medical Sciences, Beijing, China; ^4^Department of Geriatrics, Yubei District Hospital of Traditional Chinese Medicine, Chongqing, China; ^5^Department of Pharmacy, Heilongjiang University of Chinese Medicine, Harbin, China

**Keywords:** resveratrol, postmenopausal women, bone metabolism, systematic review, meta-analysis

## Abstract

**Objectives:**

This meta-analysis aims to systematically evaluate the impact of resveratrol on postmenopausal women.

**Methods:**

We searched seven electronic databases and conducted meta-analyses using Stata 12.0.

**Results:**

Ten randomized controlled trials (RCTs) with 928 participants were identified. Resveratrol significantly reduced pain scores (WMD: −2.841, 95% CI: −5.631 to −0.050, p = 0.046), pain VAS scores (WMD: −7.585, 95% CI: −12.912 to −2.257, p = 0.005), PPI scores (WMD: −8.563, 95% CI: −12.866 to −4.261, p < 0.001), and CTX levels (WMD: −0.137, 95% CI: −0.204 to −0.070, p < 0.001). However, no significant effects were observed on cognition and memory (e.g., PVT, ORR, PSM, RAVLT, LSWM, FSS, DCCS, FICA, TMT), mood (depression, overall mood), metabolic parameters (glucose, insulin, HOMA-IR, triglycerides, total cholesterol, LDL-C, HDL-C), blood pressure, sleep disturbance, menopausal symptoms, SF-36 quality of life, or bone markers ALP and OC.

**Conclusion:**

Resveratrol may improve pain and bone metabolism (CTX) in postmenopausal women but did not affect other examined outcomes. Future large-scale trials are needed to confirm these findings and determine optimal dosing and treatment strategies.

**Systematic Review Registration:**

https://www.crd.york.ac.uk/PROSPERO/#myprospero, identifier CRD42024566807.

## Graphical Abstract

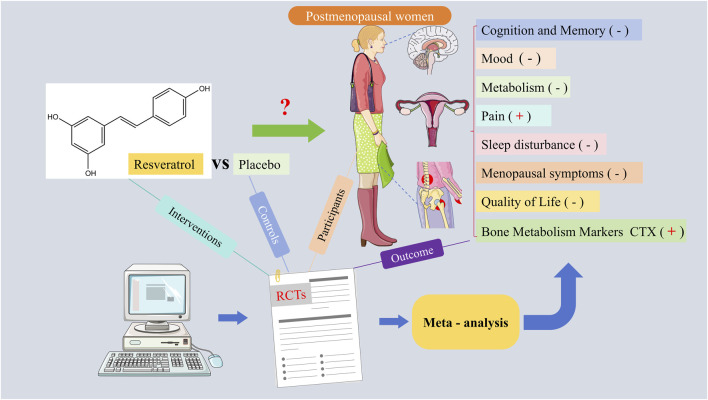

## 1 Introduction

**FIGURE 1:**
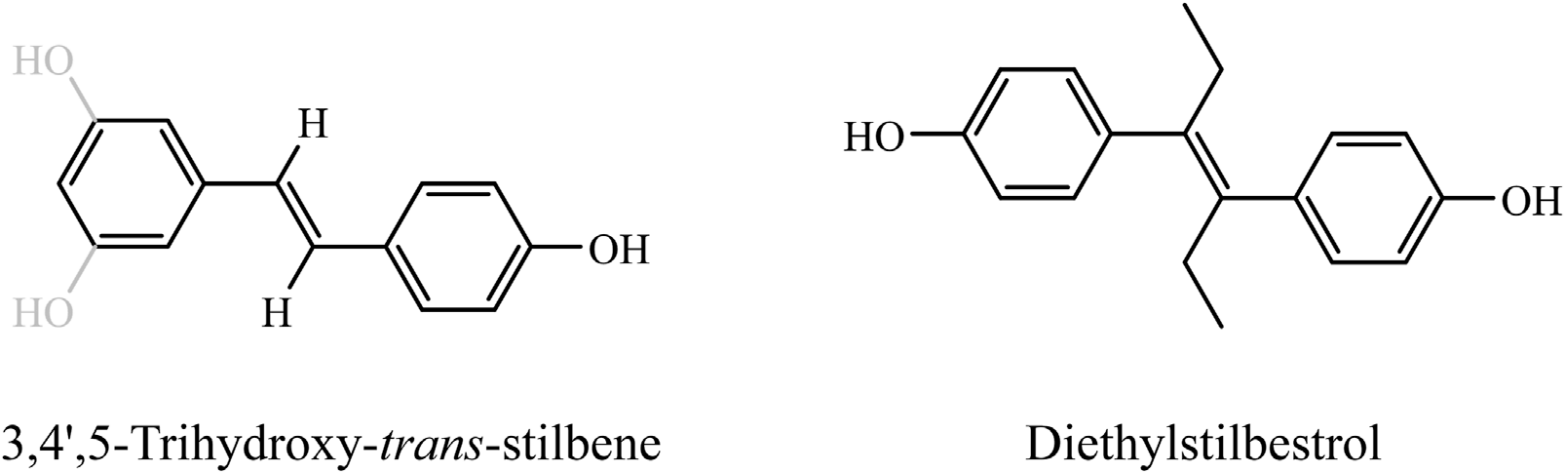
Chemical structure of resveratrol and its structural analogy to synthetic estrogen. This figure demonstrates the structural similarity between resveratrol (left) and the synthetic estrogen ethinyl estradiol (right). Both of these compounds are displayed in the trans structure. It is easy to see that they share a common vinyl diphenyl nucleus and both have hydroxyl substitutions on the benzene ring that are critical for SERM-like activity.

Resveratrol, a polyphenolic phytoalexin in the stilbene family, is commonly found in grape skins, red wine, peanuts, and certain berries (Kaur et al., 2022). Its chemical structure, consisting of two phenol rings connected by a styrene double bond and forming 3,4′,5-trihydroxy-trans-stilbene (Figure 1 left), which can exist in cis- or trans-isomeric forms, is similar to the synthetic estrogen diethylstilbestrol (Figure 1 right) (Ko et al., 2017; Tanwar et al., 2021). Although resveratrol lacks a steroid backbone, experimental studies have demonstrated its ability to bind to estrogen receptors (ERα and ERβ) and modulate estrogen-responsive genes, thereby exerting selective estrogen receptor modulator (SERM)-like activity (Qasem, 2020). The unique structure of resveratrol endows it with a variety of biological effects, including antioxidant, anti-inflammatory, anticancer, metabolic regulatory, cardiovascular protective, and bone density-enhancing properties. Consequently, resveratrol has shown positive impacts on the prevention and treatment of cardiovascular diseases, cancer, diabetes, and neurodegenerative diseases (Zhang et al., 2021). Furthermore, the potential benefits of resveratrol for postmenopausal women have attracted significant attention.

By 2035, the global population of postmenopausal women is projected to exceed 1.2 billion, accounting for 15% of the world’s female population (Zhang et al., 2025). Due to elevated follicle-stimulating hormone levels and significantly reduced estrogen levels, postmenopausal women face a variety of health challenges, including an increased risk of osteoporosis, mental health symptoms, cognitive decline, cardiovascular disease, and metabolic syndrome, all of which pose a substantial medical burden to both individuals and society (Comparetto and Borruto, 2023; Rysz et al., 2020). A meta-analysis has shown that postmenopausal women perform worse on tests of verbal memory and executive function compared to perimenopausal women (Weber et al., 2014; Zhu et al., 2022). Additionally, studies indicate that postmenopausal women are at a higher risk of cardiovascular disease than their male counterparts or younger women (Kamińska et al., 2023). This is because estrogen acts on endothelial α and β estrogen receptors to promote nitric oxide-mediated vasodilation, and the reduction of circulating estrogen post-menopause negatively impacts microcirculation, leading to accelerated arterial stiffening, reduced tissue perfusion, and decreased function of tissues and organs (Pacheco et al., 2022; Thaung Zaw et al., 2021). Moreover, oxidative stress has been identified as a key factor in the development of age-related diseases, with more pronounced effects in postmenopausal women and elderly patients, particularly in relation to cerebrovascular changes and aging-associated symptoms (Santos et al., 2024). While traditional hormone replacement therapy has proven effective in alleviating some postmenopausal symptoms, its side effects and potential risks limit its widespread use. This has led to an increasing demand in the scientific community for non-hormonal interventions. Resveratrol, as a natural compound with estrogen-like activity and antioxidant properties, has emerged as one of the alternative options due to its safety and multiple physiological effects.

Tu’s study demonstrated that resveratrol can improve cognitive function in postmenopausal women (Korovila et al., 2017), while Wong’s research showed that resveratrol can enhance bone mineral density and reduce the levels of the bone metabolism marker C-terminal telopeptide type 1 collagen (CTX) in postmenopausal women (Meyer et al., 2024). Additionally, resveratrol has been shown to improve mood, cerebrovascular function, metabolic markers, and menopausal symptoms in peri- and postmenopausal women (Tu et al., 2023; Wong et al., 2020). Conversely, study have reported that resveratrol does not improve cognition or metabolism in menopausal women (Evans et al., 2017). These contradictory findings highlight the need for systematic reviews and meta-analyses, which occupy the highest level of evidence in the hierarchy (Thaung Zaw et al., 2020a). Therefore, to provide reliable evidence on the efficacy of resveratrol supplementation in postmenopausal women, we aim to conduct a systematic review and meta-analysis to comprehensively evaluate the existing randomized controlled trials (RCTs) and perform a quantitative analysis of the available data. This will help assess the impact of resveratrol on postmenopausal women and provide insights for future research and clinical applications.

## 2 Methods

### 2.1 Protocol

This systematic review was conducted following the guidelines outlined in the Cochrane Handbook for Systematic Reviews of Interventions (version 6.2) and reported according to the Preferred Reporting Items for Systematic Reviews and Meta-Analyses (PRISMA) guidelines, and the protocol was registered on PROSPERO (CRD42024566807). [https://www.crd.york.ac.uk/PROSPERO/#myprospero].

### 2.2 Search strategy

We systematically searched electronic databases, including PubMed, Embase, Cochrane Library, China National Knowledge Infrastructure, Wanfang Data, and Chinese VIP Information for studies from inception to January 2025. No language restrictions were applied. Search terms included “Resveratrol,” “trans-resveratrol,” “postmenopausal women,” “senile women,” “female,” “women,” “random controlled trial,” “random,” “randomized,” “controlled,” and “RCT.” Full details of the search strategy are available in Supplementary File S1. We also manually searched reference lists of previously published systematic reviews and meta-analyses on this topic to identify further eligible studies. The search was independently conducted by two authors (WW and TM).

### 2.3 Inclusion and exclusion criteria

#### 2.3.1 Inclusion criteria

(1) Participants: Postmenopausal women;
(2) Interventions: Resveratrol in various formulations;
(3) Controls: Placebo;
(4) Outcome measures: ①Outcome measures assessing cognition and memory: Pattern comparison speed test (PCT), the trail making task A (TMT A), picture vocabulary test (PVT), oral reading recognition test (ORR), picture sequence memory test (PSM), the rey auditory verbal learning test (RAVLT) immediate, RAVLT delayed, list sorting working memory test (LSWM), forward spatial span test (FSS), dimensional change card sort test (DCCS), flanker inhibitory control and attention test (FICA), and TMT performance; ②Outcome measures assessing mood: Depression, the centre for epidemiologic studies depression scale (CES-D), overall mood; ③Outcome measures assessing metabolism: glucose, insulin, homeostatic model assessment of insulin resistance (HOMA-IR), triglycerides, total cholesterol, low density lipoprotein (LDL)-cholesterol, high density lipoprotein (HDL)-cholesterol, systolic blood pressure (BP), and diastolic BP; ④Outcome measures assessing pain: Pain scores, pain visual analog scale (VAS) scores, present pain intensity (PPI) scores; ⑤Sleep disturbance; ⑥Menopausal symptoms: psychological, somatic, and urogenital menopausal symptoms; ⑦the shortform (SF)-36 quality-of-life; ⑧Bone metabolic markers: CTX, alkaline phosphatase (ALP), and osteocalcin (OC);
(5) Study design: Randomized controlled trials (RCTs).

#### 2.3.2 Exclusion criteria

(1) Overlapping data;
(2) Cross-sectional studies, reviews, abstracts, study protocols, conference papers, or papers not reporting any outcomes of interest.

Two independent authors selected studies based on these criteria (FJ and JL). After removing duplicates, they screened titles/abstracts and full texts to determine eligible studies. Discrepancies were resolved through discussion with a third person (YZ).

### 2.4 Data extraction

Two investigators (JH and GZ) independently reviewed and extracted the following information: first author, year of publication, region, types of included literature, sample size, age, course of disease, type and duration of intervention, and outcomes. For missing data, we contacted the authors.

### 2.5 Risk of bias assessment

The quality of the included studies was assessed using the Cochrane Collaboration’s tool for assessing the risk of bias. Assessment parameters included random sequence generation (selection bias), allocation concealment (selection bias), blinding of participants and personnel (performance bias), blinding of outcome assessment (detection bias), incomplete outcome data (attrition bias), selective reporting (reporting bias), and other biases. According to the Cochrane Handbook’s recommendations, a judgment of “Yes” indicated a low risk of bias, “No” indicated a high risk of bias, and “Unclear” indicated an unclear or unknown risk of bias. The risk of bias for included studies was independently assessed by two authors (MY and YZ), with disagreements resolved through discussion with a third assessor (WW).

### 2.6 Statistical analysis

We use the Stata software (version 12.0; StataCorp, College Station, TX) to conduct statistical analysis. The weighted mean difference (WMD) for continuous variables with 95% confidence intervals (CIs) was used. The mean changes and SD were used to calculate WMD. If mean changes were not reported directly, calculated by subtraction value levels at baseline from the end of the intervention. Also, the SD was estimated by using the following formula from the Cochrane Handbook: SD = SE × √n. Heterogeneity was assessed by the Q test and the *I* ^2^ statistic. When p ≥ 0.10 and *I* ^2^ ≤ 50%, the fixed effect model was used; p ≤ 0.10 and *I* ^2^ ≥50%, the random effects model was used. When the p ≥ 0.10 and *I* ^2^ ≥ 50%, the number of studies included in the meta-analysis will be considered. The fixed effect model will be used if the number of studies is less than 5; otherwise, the random-effects model will be applied (Tufanaru et al., 2015). P ≤ 0.05 was considered statistically significant. The publication bias was assessed by funnel plots and Egger’s test. When significant heterogeneity was detected, the sensitivity analysis was conducted to assess the stability of the results by excluding individual studies one by one.

## 3 Results

### 3.1 Literature search

**FIGURE 2:**
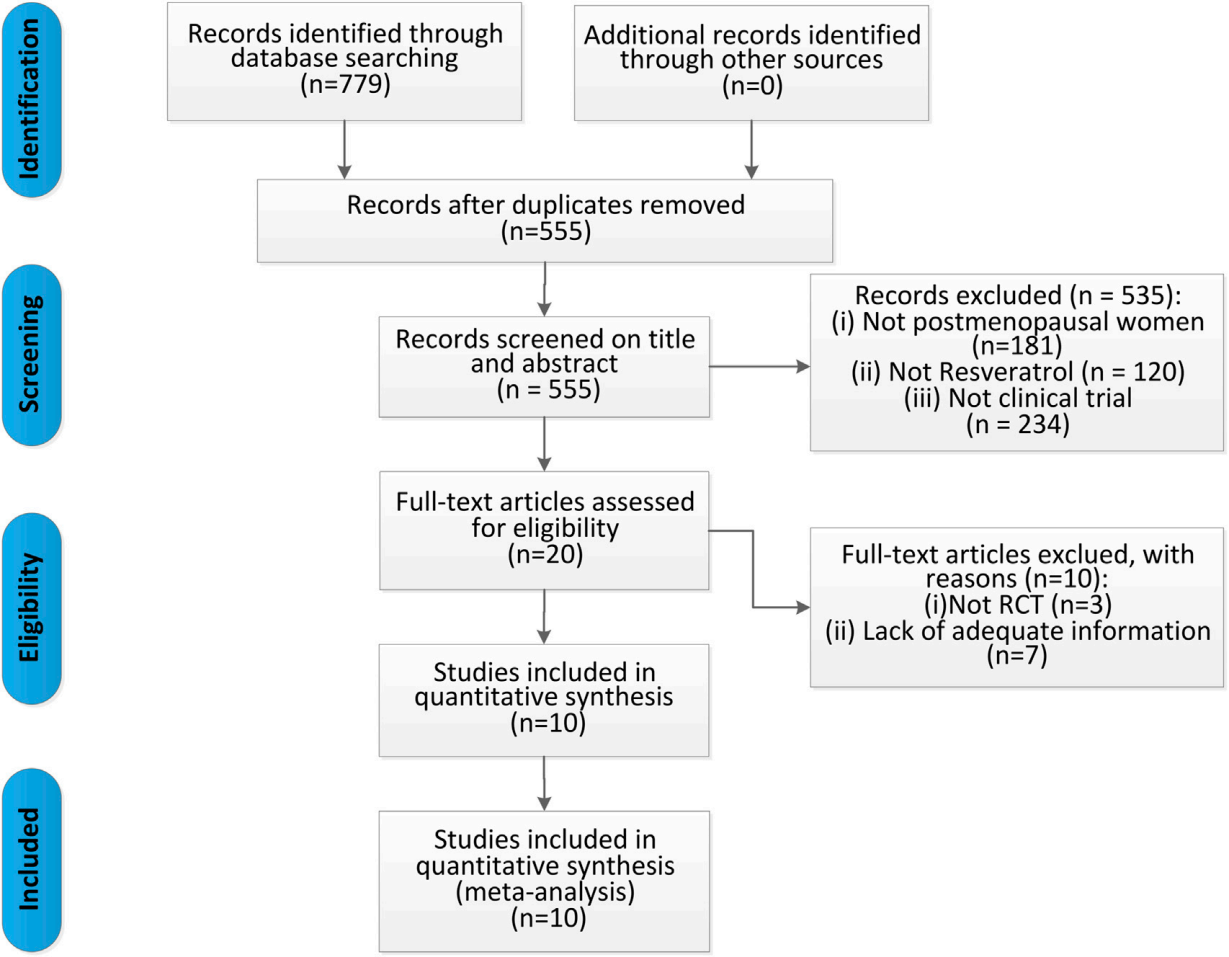
Flow diagram of studies selection process.

Initially, we identified 779 records through database searching. No additional records were identified through other sources. After removing duplicates, 555 records remained. Screening titles and abstracts led to the exclusion of 535 records that did not meet the inclusion criteria (Not postmenopausal women, not resveratrol and not clinical trial). We then conducted a full-text review of the remaining 20 articles, excluding 10 due to: (1) not RCT and (2) lack of adequate information. Finally, 10 articles (Evans et al., 2017; Ozemek et al., 2020; Prickett et al., 2023; Thaung Zaw et al., 2021; Thaung Zaw et al., 2020a; Thaung Zaw et al., 2020b; Wong et al., 2020; Wong et al., 2017; Yoshino et al., 2012; Jiang et al., 2019) were included in our meta-analysis, as detailed in Figure 2.

### 3.2 Study characteristics and quality assessment

Table 1 summarizes the characteristics of the included studies. From 2012 to 2023, 10 RCTs were conducted: 6 in Australia (Evans et al., 2017; Thaung Zaw et al., 2021; Thaung Zaw et al., 2020a; Thaung Zaw et al., 2020b; Wong et al., 2020; Wong et al., 2017), 2 in the America (Ozemek et al., 2020; Yoshino et al., 2012), 1 in New Zealand (Prickett et al., 2023), and 1 in China (Jiang et al., 2019). Nine of them were published in English (Evans et al., 2017; Ozemek et al., 2020; Prickett et al., 2023; Thaung Zaw et al., 2021; Thaung Zaw et al., 2020a; Thaung Zaw et al., 2020b; Wong et al., 2020; Wong et al., 2017; Yoshino et al., 2012), and one was in Chinese (Jiang et al., 2019). These studies involved 928 participants, with sample sizes ranging from 15 to 146 per study. In the experimental groups, 5 studies (Evans et al., 2017; Prickett et al., 2023; Wong et al., 2020; Yoshino et al., 2012; Jiang et al., 2019) used resveratrol treatment, and the remaining 5 (Ozemek et al., 2020; Thaung Zaw et al., 2021; Thaung Zaw et al., 2020a; Thaung Zaw et al., 2020b; Wong et al., 2017) used trans-resveratrol treatment. All control groups received placebo treatment. The treatment duration ranged from 14 weeks to 12 months. The study outcomes were: 3 studies (Evans et al., 2017; Thaung Zaw et al., 2021; Thaung Zaw et al., 2020b) reported RAVLT immediate, RAVLT delayed and TMT A, 1 study (Evans et al., 2017) reported CES-D, 2 studies (Thaung Zaw et al., 2021; Thaung Zaw et al., 2020b) reported PCT, PVT, ORR, PSM, LSWM, FSS, DCCS, FICA, and TMT performance. Four studies (Ozemek et al., 2020; Thaung Zaw et al., 2021; Thaung Zaw et al., 2020b; Yoshino et al., 2012) reported glucose, insulin, LDL-cholesterol, and HDL-cholesterol, 3 studies (Thaung Zaw et al., 2021; Thaung Zaw et al., 2020b; Yoshino et al., 2012) reported HOMA-IR and triglycerides, 3 studies (Ozemek et al., 2020; Thaung Zaw et al., 2021; Yoshino et al., 2012) reported systolic and diastolic BP, 2 studies (Thaung Zaw et al., 2020a; Wong et al., 2017) reported pain scores, pain VAS scores, PPI scores, sleep disturbance, menopausal symptoms (psychological, somatic, and urogenital menopausal symptoms), SF-36 quality-of-life, and overall mood, 3 studies (Evans et al., 2017; Thaung Zaw et al., 2020a; Wong et al., 2017) reported depression, 3 studies (Prickett et al., 2023; Wong et al., 2020; Jiang et al., 2019) reported CTX and OC, and 2 studies (Prickett et al., 2023; Jiang et al., 2019) reported ALP.

**TABLE 1:** Characteristics of the included studies.

Study	Region	Types	Sample size (TG/CG)	Included population	Age (Y)		Interventions		Duration	Outcomes
					TG	CG	TG	CG		
Evans et al. (2017)	Australia	RCT, double-blind	79 (38/41)	Postmenopausal women	61.5 ± 1.1	61.5 ± 1.2	Resveratrol (75 mg, bid)	Placebo	14 weeks	(1), (2), (3), (4), (5)
Jiang et al. (2019)	China	RCT	84 (42/42)	Postmenopausal women	56.64 ± 5.85	57.08 ± 5.82	Resveratrol (0.4 g/d)	Placebo	3 months	(6), (7), (8)
Ozemek et al. (2020)	America	RCT, double-blind	15	Postmenopausal women	58.1 ± 3.2	58.1 ± 3.2	Trans-resveratrol (250 mg/d)	Placebo	NR	(9), (10), (11), (12), (13), (14)
Prickett et al. (2023)	New Zealand	RCT, double-blind	125 (60/65)	Postmenopausal women	NR	NR	Resveratrol (75 mg/d)	Placebo	12 months	(6), (7), (8)
Thaung Zaw et al. (2020b)	Australia	RCT, double-blind	146 (73/73)	Postmenopausal women	64 ± 1	64 ± 1	Trans-resveratrol (75mg, bid)	Placebo	12 months	(1), (2), (3), (9), (10), (11), (12), (15), (16), (17), (18), (19), (20), (21), (22), (23), (24), (25), (26)
Thaung Zaw et al. (2020a)	Australia	RCT, double-blind	125	Postmenopausal women	65 ± 0.7	65 ± 0.7	Trans-resveratrol (75 mg, bid)	Placebo	12 months	(4), (27), (28), (29), (30), (31), (32), (33)
Thaung Zaw et al. (2021)	Australia	RCT, double-blind	125 (62/63)	Postmenopausal women	65 ± 7	66 ± 8	Trans-resveratrol (75 mg, bid)	Placebo	24 months	(1), (2), (3), (9), (10), (11), (12), (13), (14), (15), (16), (17), (18), (19), (20), (21), (22), (23), (24), (25), (26)
Wong et al. (2017)	Australia	RCT, double-blind	72 (37/35)	Postmenopausal women	61.3 ± 1.1	61.5 ± 1.4	Trans-resveratrol (75 mg, bid)	Placebo	14 weeks	(4), (27), (28), (29), (30), (31), (32), (33)
Wong et al. (2020)	Australia	RCT, double-blind	128 (63/65)	Postmenopausal women	65.8 ± 1.3	64.3 ± 1.3	Resveratrol (75 mg/d)	Placebo	12 months	(6), (8)
Yoshino et al. (2012)	America	RCT, double-blind	29 (15/14)	Postmenopausal women	58.2 ± 4.0	59.8 ± 4.3	Resveratrol (75 mg/d)	Placebo	12 weeks	(9), (10), (11), (12), (13), (14), (24), (25), (26)

Note. RCT, randomized controlled trial; TG, trial group; CG, control group; F, female; M, male; NR, not reported; (1): the Rey Auditory Verbal Learning Test immediate; (2) the Rey Auditory Verbal Learning Test delayed; (3): the Trail Making Task A; (4): Depression; (5): the Centre for Epidemiologic Studies Depression scales; (6): C-terminal telopeptide type 1 collagen; (7): alkaline phosphatase; (8): Osteocalcin.; (9): Glucose; (10): Insulin; (11): LDL-cholesterol; (12): HDL-cholesterol; (13): Systolic BP; (14): Diastolic BP; (15): Pattern Comparison Speed Test; (16): Picture Vocabulary Test; (17): Oral Reading Recognition Test; (18): Picture Sequence Memory Test; (19): List Sorting Working Memory Test; (20): Forward Spatial Span test; (21): Dimensional Change Card Sort Test; (22): Flanker Inhibitory Control and Attention Test; (23): the Trail Making Task performance; (24): Homeostatic Model Assessment of Insulin Resistance; (25): Triglycerides; (26):Total cholesterol; (27): Pain scores; (28): Pain visual analog scores; (29): Present pain intensity scores; (30): Sleep disturbance; (31): Menopausal symptoms; (32): SF-36, quality-of-life; (33): Overall Mood scale.

**FIGURE 3:**
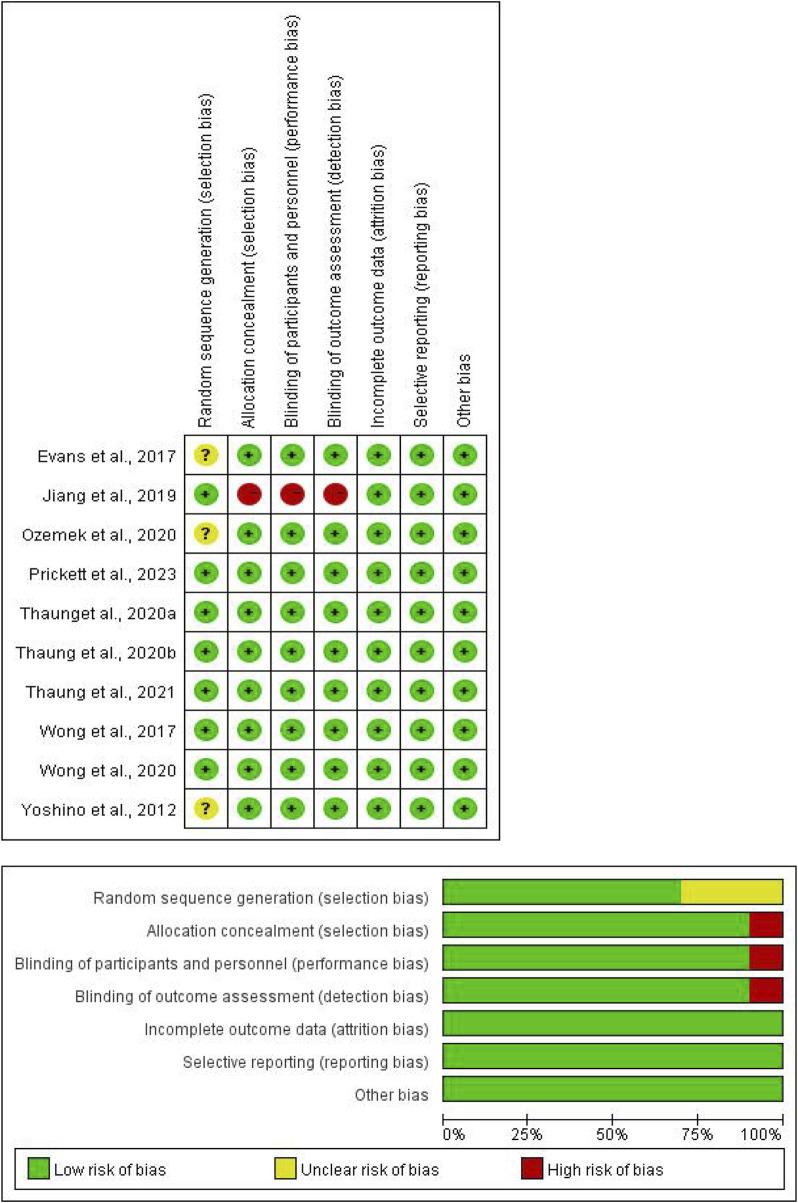
Risk of bias.

Table 2 and Figure 3 displays the quality assessment of the included studies. The Cochrane scores ranged from 4 to 7, with 6 studies (Prickett et al., 2023; Thaung Zaw et al., 2021; Thaung Zaw et al., 2020a; Thaung Zaw et al., 2020b; Wong et al., 2020; Wong et al., 2017) scoring 7, 3 studies (Evans et al., 2017; Ozemek et al., 2020; Yoshino et al., 2012) scoring 6, and 1 study (Jiang et al., 2019) scoring 4. All included studies reported random allocation, with 7 studies (Prickett et al., 2023; Thaung Zaw et al., 2021; Thaung Zaw et al., 2020a; Thaung Zaw et al., 2020b; Wong et al., 2020; Wong et al., 2017; Jiang et al., 2019) describing the random sequence generation method, while 3 studies (Evans et al., 2017; Ozemek et al., 2020; Yoshino et al., 2012) did not provide detailed information. Allocation concealment was mentioned in 9 studies (Evans et al., 2017; Ozemek et al., 2020; Prickett et al., 2023; Thaung Zaw et al., 2021; Thaung Zaw et al., 2020a; Thaung Zaw et al., 2020b; Wong et al., 2020; Wong et al., 2017; Yoshino et al., 2012), but not in 1 study (Jiang et al., 2019). Double-blinding of participants and outcome assessors was reported in 9 trials (Evans et al., 2017; Ozemek et al., 2020; Prickett et al., 2023; Thaung Zaw et al., 2021; Thaung Zaw et al., 2020a; Thaung Zaw et al., 2020b; Wong et al., 2020; Wong et al., 2017; Yoshino et al., 2012), with 1 study (Jiang et al., 2019) not mentioning it. All studies met the criteria for incomplete outcome data and reported prespecified outcomes, with no other biases identified.

**TABLE 2:** Quality assessment of included studies based on Cochrane guidelines.

Study	Random sequence generation	Allocation concealment	Blinding of participants and personnel	Blinding of outcome assessment	Incomplete outcome data	Selective reporting	Free of other bias	Total score
Evans et al. (2017)	Unclear	Low risk	Low risk	Low risk	Low risk	Low risk	Low risk	6
Jiang et al. (2019)	Low risk	High risk	High risk	High risk	Low risk	Low risk	Low risk	4
Ozemek et al. (2020)	Unclear	Low risk	Low risk	Low risk	Low risk	Low risk	Low risk	6
Prickett et al. (2023)	Low risk	Low risk	Low risk	Low risk	Low risk	Low risk	Low risk	7
Thaung Zaw et al. (2020b)	Low risk	Low risk	Low risk	Low risk	Low risk	Low risk	Low risk	7
Thaung Zaw et al. (2020a)	Low risk	Low risk	Low risk	Low risk	Low risk	Low risk	Low risk	7
Thaung Zaw et al. (2021)	Low risk	Low risk	Low risk	Low risk	Low risk	Low risk	Low risk	7
Wong et al. (2017)	Low risk	Low risk	Low risk	Low risk	Low risk	Low risk	Low risk	7
Wong et al. (2020)	Low risk	Low risk	Low risk	Low risk	Low risk	Low risk	Low risk	7
Yoshino et al. (2012)	Unclear	Low risk	Low risk	Low risk	Low risk	Low risk	Low risk	6

### 3.3 Results of meta-analysis

#### 3.3.1 Effects of resveratrol on cognition and memory

##### 3.3.1.1 Processing speed

**FIGURE 4:**
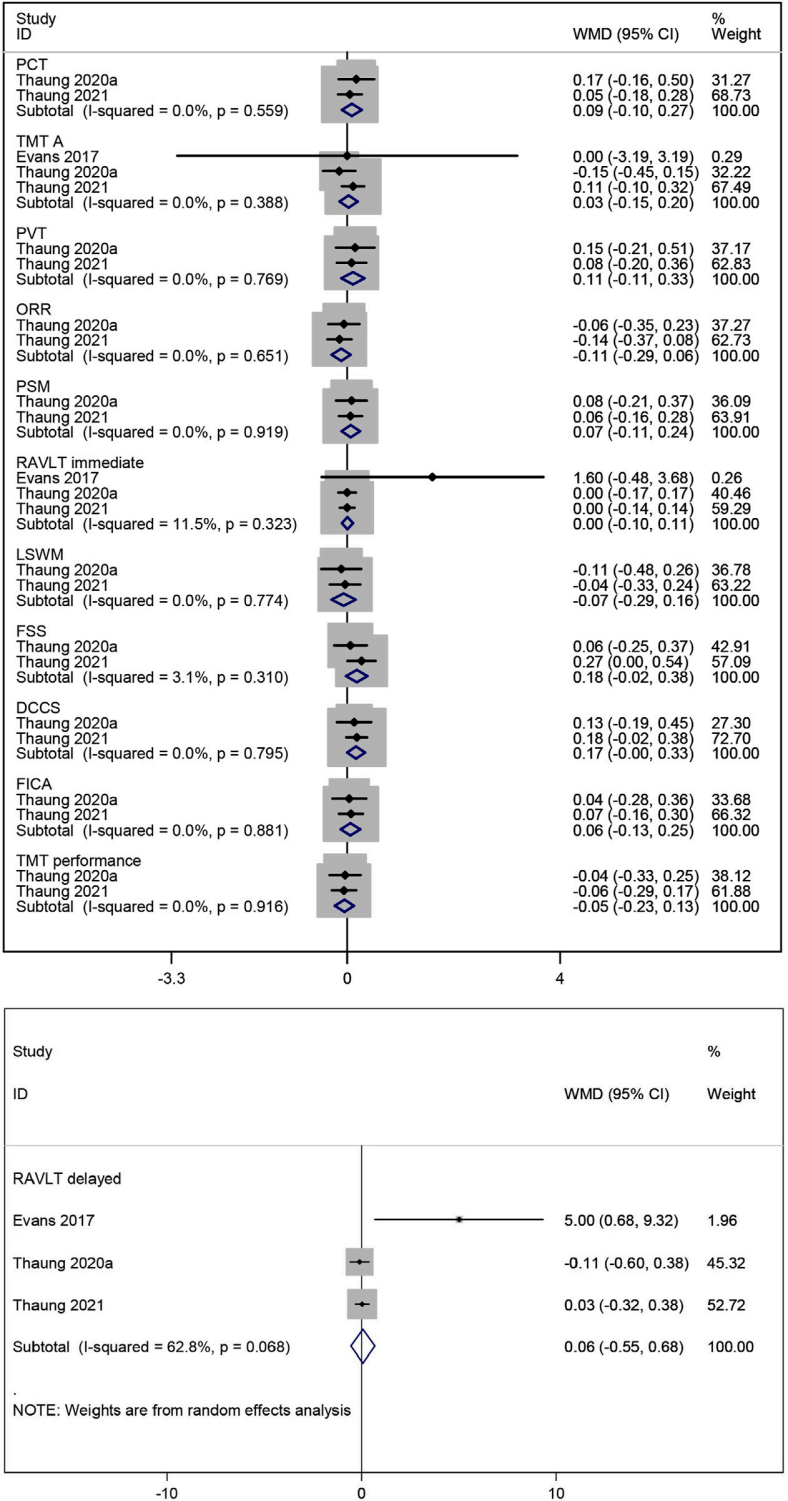
Forest plot for PCT, TMT A, PVT, ORR, PSM, RAVLT immediate, LSWM, FSS, DCCS, FICA, TMT performance and RAVLT delayed.

The effect of resveratrol on processing speed in postmenopausal women was assessed using two measures (PCT and TMT A). A meta-analysis of two studies (Thaung Zaw et al., 2021; Thaung Zaw et al., 2020b) showed that resveratrol had no statistically significant effect on PCT compared to placebo (WMD: 0.088, 95% CI: −0.099 to 0.274, p = 0.358; Figure 4). Combined data from three studies (Evans et al., 2017; Thaung Zaw et al., 2021; Thaung Zaw et al., 2020b) on TMT A also indicated no significant effect of resveratrol treatment (WMD: 0.026, 95% CI: −0.147 to 0.199, p = 0.769; Figure 4).

##### 3.3.1.2 Language

The impact of resveratrol on language ability in postmenopausal women was evaluated using two measures (PVT and ORR). Meta-analysis of two studies (Thaung Zaw et al., 2021; Thaung Zaw et al., 2020b) reported that resveratrol did not improve language ability compared to placebo (WMD: 0.107, 95% CI: −0.112 to 0.327, p = 0.339; WMD: −0.113, 95% CI: −0.291 to 0.065, p = 0.212; respectively; Figure 4).

##### 3.3.1.3 Episodic memory

PSM was used to evaluate the effect of resveratrol on episodic memory in postmenopausal women. Combined data from two studies (Thaung Zaw et al., 2021; Thaung Zaw et al., 2020b) showed no statistically significant improvement in episodic memory with resveratrol compared to placebo (WMD: 0.068, 95% CI: −0.107 to 0.243, p = 0.447; Figure 4).

##### 3.3.1.4 Verbal memory

The effect of resveratrol on verbal memory in postmenopausal women was assessed using two measures (RAVLT immediate and RAVLT delayed). The combined results of three studies (Evans et al., 2017; Thaung Zaw et al., 2021; Thaung Zaw et al., 2020b) on them indicated no significant improvement with resveratrol compared to placebo (WMD: 0.004, 95% CI: −0.102 to 0.110, p = 0.939; WMD: 0.064, 95% CI: −0.550 to 0.678, p = 0.838; respectively; Figure 4).

##### 3.3.1.5 Working memory

The effect of resveratrol on working memory in postmenopausal women was evaluated using two measures (LSWM and FSS). Meta-analysis of two studies (Thaung Zaw et al., 2021; Thaung Zaw et al., 2020b) showed no significant improvement in working memory with resveratrol compared to placebo (WMD: −0.066, 95% CI: −0.293 to 0.161, p = 0.567; WMD: 0.180, 95% CI: −0.021 to 0.380, p = 0.079; respectively; Figure 4).

##### 3.3.1.6 Cognitive flexibility

The effect of resveratrol on cognitive flexibility in postmenopausal women was assessed using three measures (DCCS, FICA, and TMT performance). Combined data of two studies (Thaung Zaw et al., 2021; Thaung Zaw et al., 2020b) indicated no significant improvement in cognitive flexibility with resveratrol compared to placebo (WMD: 0.166, 95% CI: −0.002 to 0.334, p = 0.052; WMD: 0.060, 95% CI: −0.125 to 0.245, p = 0.526; WMD: −0.052, 95% CI: −0.232 to 0.128, p = 0.568; respectively; Figure 4).

#### 3.3.2 Effects of resveratrol on mood

**FIGURE 5:**
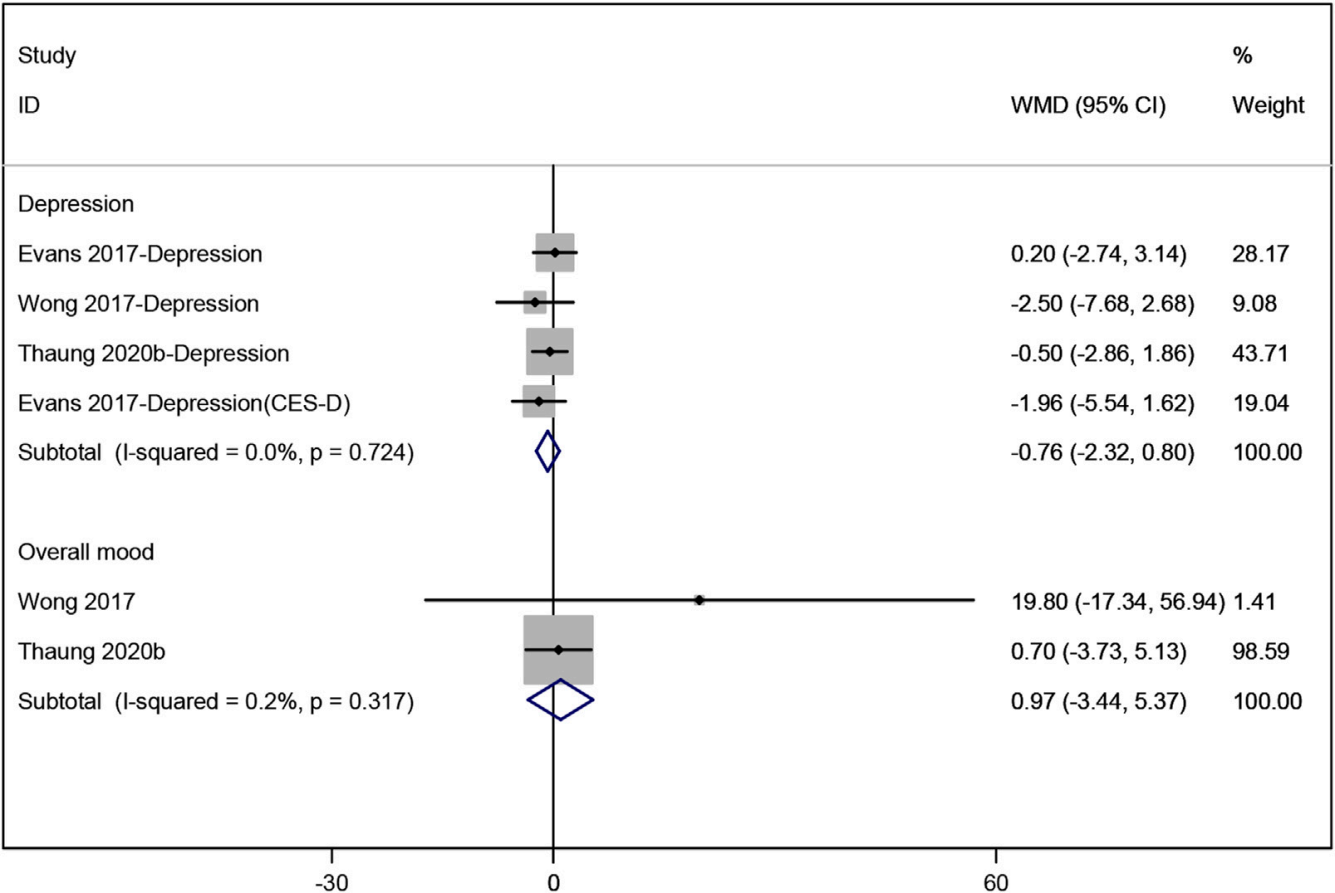
Forest plot for depression and overall mood.

Three studies (Evans et al., 2017; Thaung Zaw et al., 2020a; Wong et al., 2017) evaluated the effect of resveratrol on depression, with the pooled data showing no significant change (WMD: −0.762, 95% CI: −2.323 to 0.798, p = 0.338; Figure 5). Similarly, a meta-analysis of two studies (Thaung Zaw et al., 2020a; Wong et al., 2017) assessing overall mood showed no statistically significant results (WMD: 0.968, 95% CI: −3.435 to 5.372, p = 0.666; Figure 5).

#### 3.3.3 Effects of resveratrol on metabolism

**FIGURE 6:**
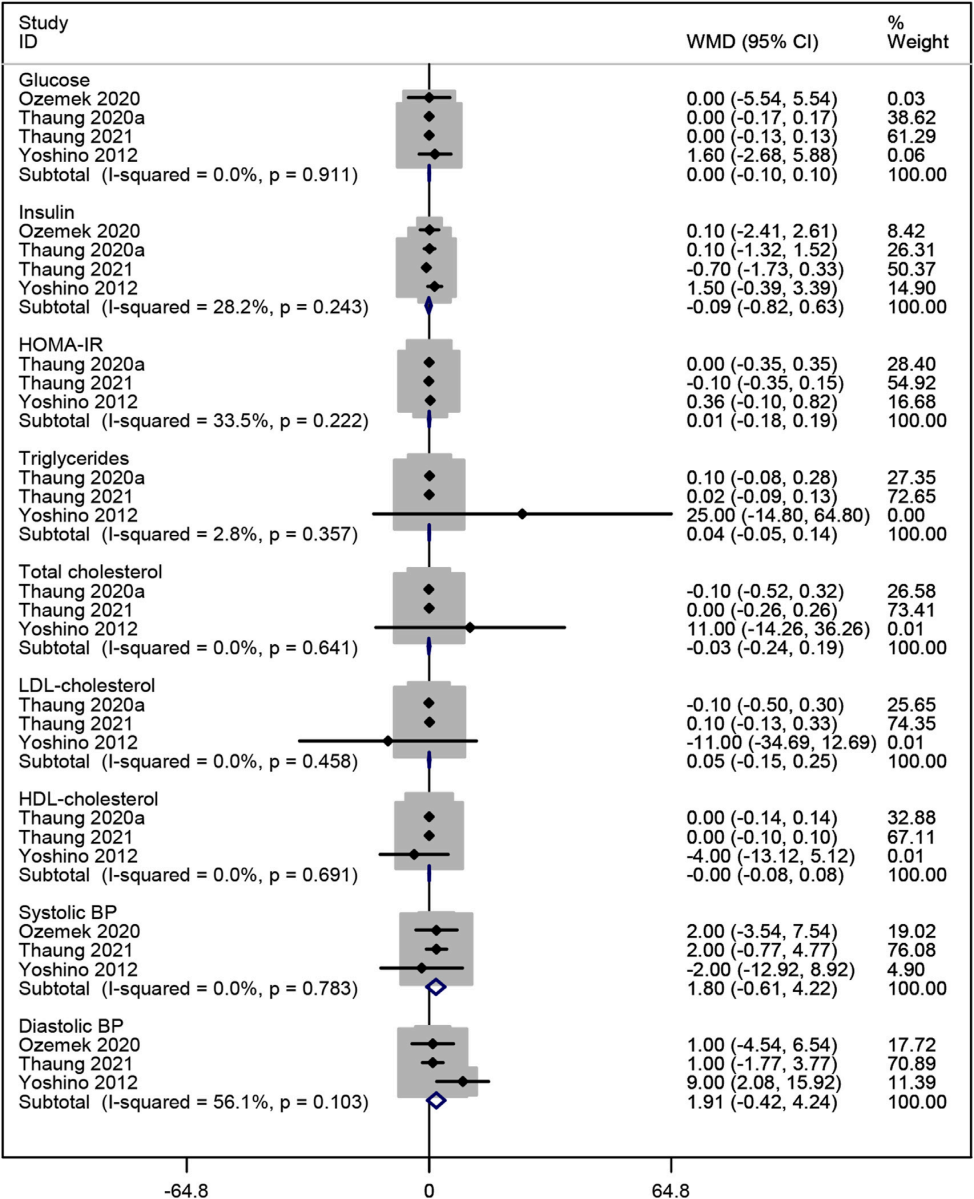
Forest plot for glucose, insulin, HOMA-IR, triglycerides, total cholesterol, LDL-cholesterol, HDL-cholesterol, systolic BP and diastolic BP.

Four studies (Ozemek et al., 2020; Thaung Zaw et al., 2021; Thaung Zaw et al., 2020b; Yoshino et al., 2012) analyzed the effects of resveratrol on glucose and insulin levels. The pooled data revealed no significant improvement in glucose or insulin levels compared to placebo (WMD: 0.001, 95% CI: −0.102 to 0.104, p = 0.986; WMD: −0.094, 95% CI: −0.823 to 0.634, p = 0.799; respectively; Figure 6). Additionally, three studies (Thaung Zaw et al., 2021; Thaung Zaw et al., 2020b; Yoshino et al., 2012) reported no significant effect of resveratrol on HOMA-IR, triglycerides, total cholesterol, LDL-cholesterol, and HDL-cholesterol (WMD: 0.005, 95% CI: −0.181 to 0.191, p = 0.957; WMD: 0.042, 95% CI: −0.052 to 0.137, p = 0.383; WMD: −0.026, 95% CI: −0.244 to 0.193, p = 0.817; WMD: 0.048, 95% CI: −0.153 to 0.249, p = 0.640; WMD: −0.000, 95% CI: −0.080 to 0.079, p = 0.994; respectively; Figure 6). Three studies (Ozemek et al., 2020; Thaung Zaw et al., 2021; Yoshino et al., 2012) evaluated the effects on BP, showing no significant reduction in systolic or diastolic BP compared to placebo (WMD: 1.804, 95% CI: −0.614 to 4.222, p = 0.144; WMD: 1.911, 95% CI: −0.423 to 4.245, p = 0.109; respectively; Figure 6).

#### 3.3.4 Effects of resveratrol on pain

**FIGURE 7:**
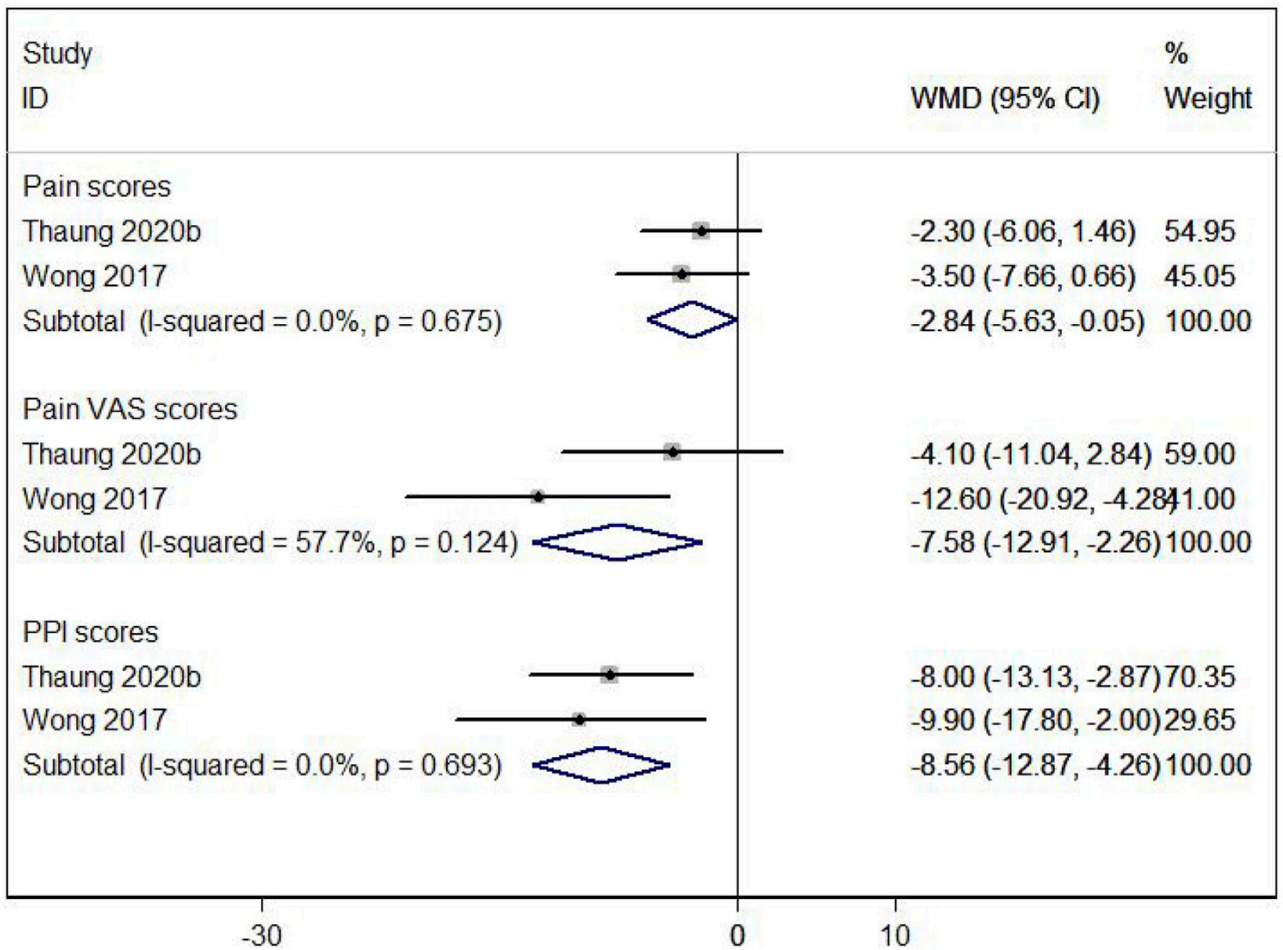
Forest plot for pain scores, pain VAS scores and PPl scores.

The impact of resveratrol on pain in postmenopausal women was assessed using three measures: pain scores, pain VAS scores, and PPI scores. Two studies (Thaung Zaw et al., 2020a; Wong et al., 2017) showed that resveratrol significantly improved these pain measures compared to placebo (WMD: −2.841, 95% CI: −5.631 to −0.050, p = 0.046; WMD: −7.585, 95% CI: −12.912 to −2.257, p = 0.005; WMD: −8.563, 95% CI: −12.866 to −4.261, p = 0.000; respectively; Figure 7).

#### 3.3.5 Effects of resveratrol on sleep disturbance

**FIGURE 8:**
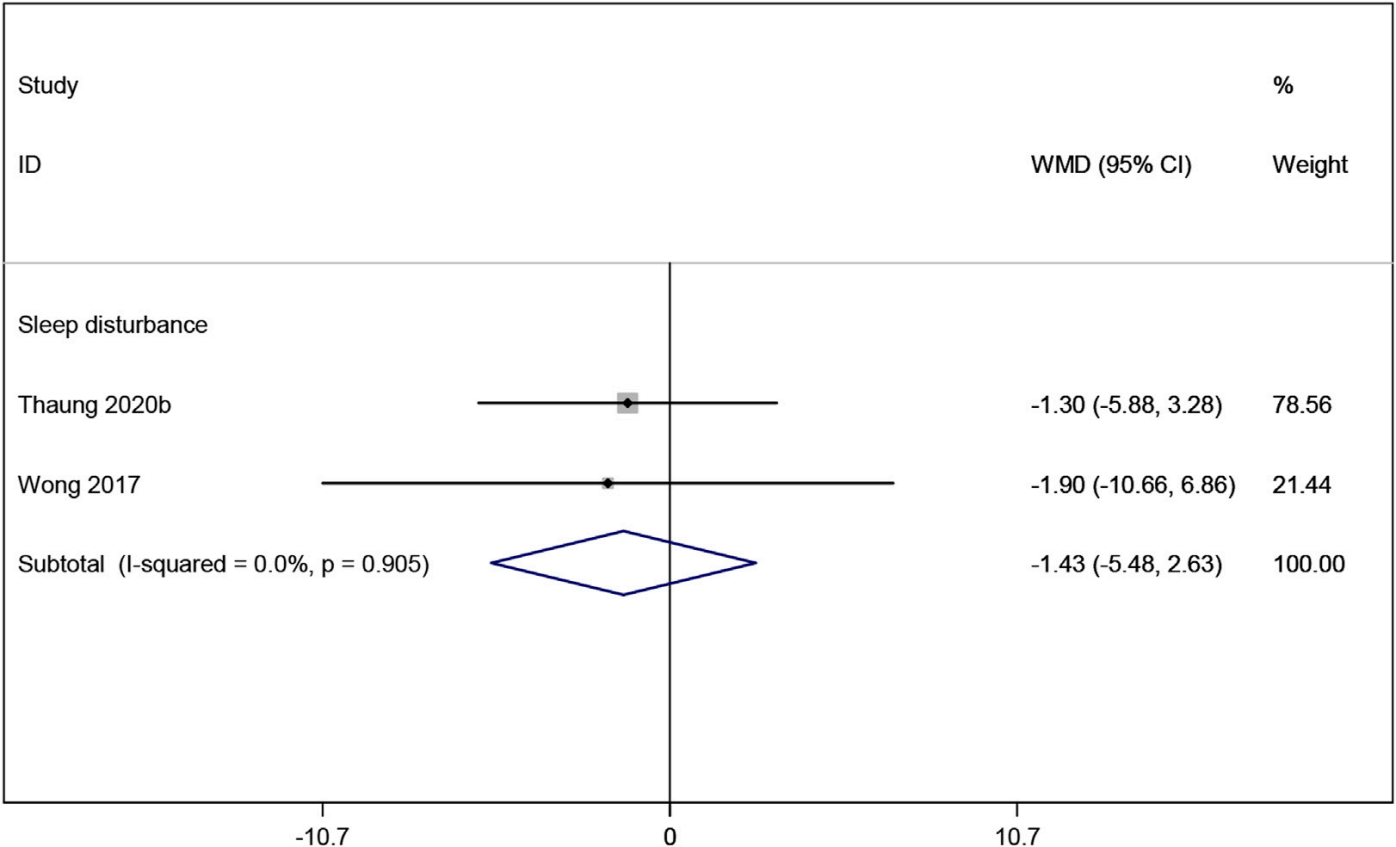
Forest plot for sleep disturbance.

Two studies (Thaung Zaw et al., 2020a; Wong et al., 2017) reported sleep disturbance, and the meta-analysis results indicating no significant improvement with resveratrol compared to placebo (WMD: −1.429, 95% CI: −5.484 to 2.627, p = 0.490; Figure 8). The sleep disturbance outcome was assessed using the Pittsburgh Sleep Quality Index (PSQI), a validated measure widely used to assess sleep quality and disturbances (Buysse et al., 1989).

#### 3.3.6 Effects of resveratrol on menopausal symptoms

**FIGURE 9:**
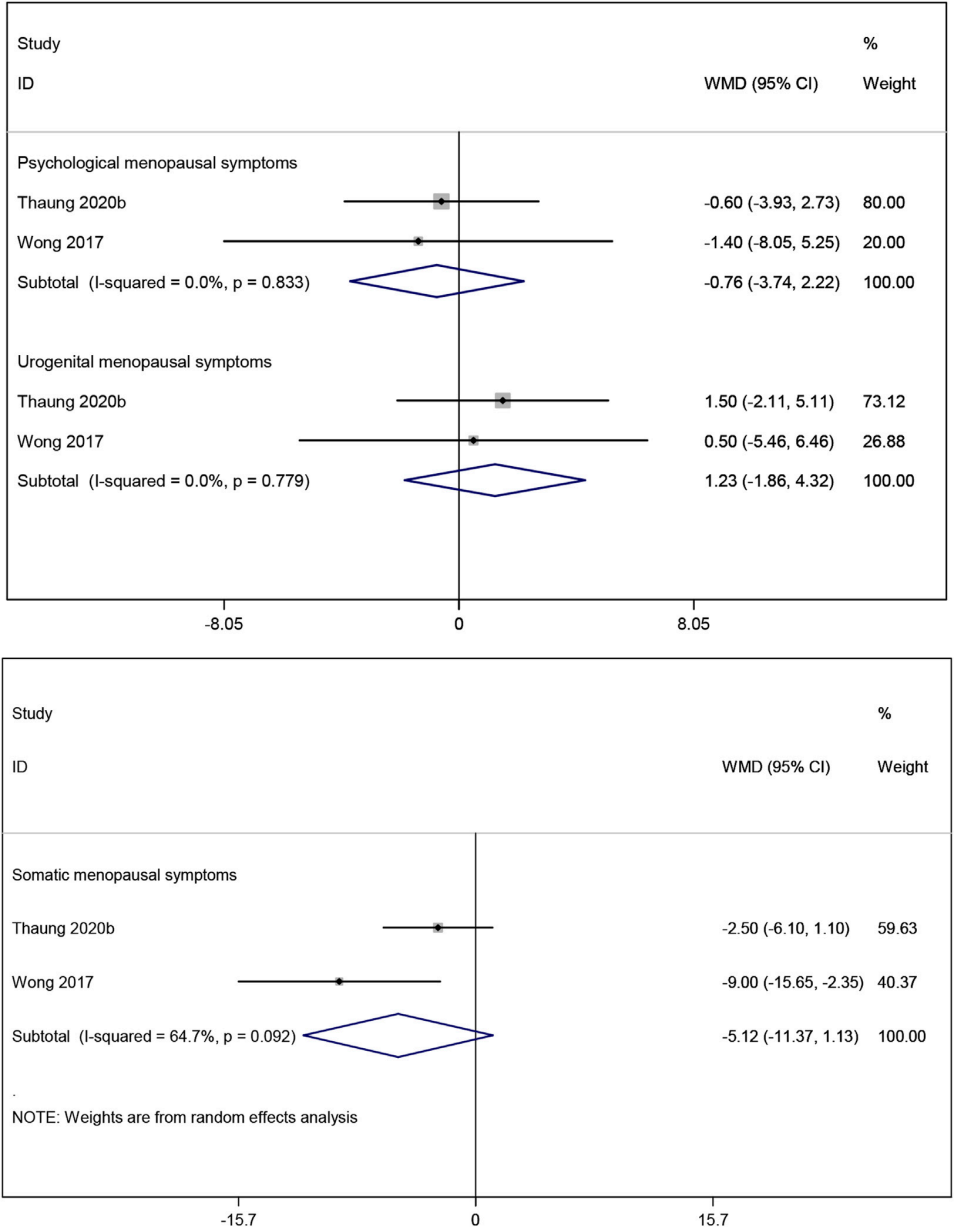
Forest plot for psychological menopausal symptoms, urogenital menopausal symptoms and somatic menopausal symptoms.

The result of meta-analysis two studies (Thaung Zaw et al., 2020a; Wong et al., 2017) showed that resveratrol had no statistically significant effect on psychological, somatic and urogenital menopausal symptoms (WMD: −0.760, 95% CI: −3.735 to 2.215, p = 0.617; WMD: −5.124, 95% CI: −11.374 to 1.127, p = 0.108; WMD: 1.231, 95% CI: −1.859 to 4.322, p = 0.435; respectively; Figure 9).

#### 3.3.7 Effects of resveratrol on quality of life

**FIGURE 10:**
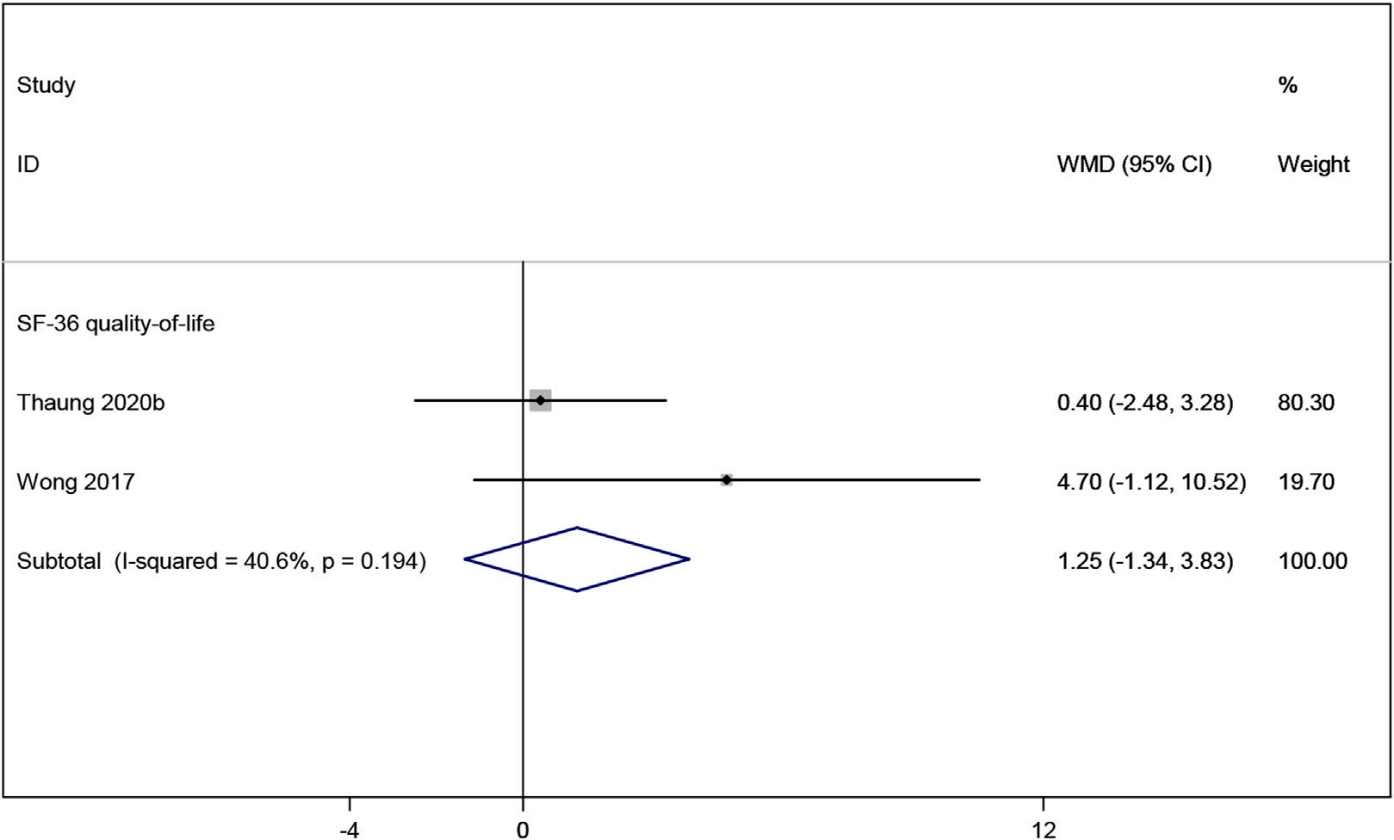
Forest plot for SF-36 quality-of-life.

Two studies (Thaung Zaw et al., 2020a; Wong et al., 2017) reported the SF-36 quality-of-life measure, which indicated no significant improvement in quality of life in postmenopausal women taking resveratrol compared to placebo (WMD: 1.247, 95% CI: −1.336 to 3.830, p = 0.344; Figure 10).

#### 3.3.8 Effects of resveratrol on bone metabolism markers

**FIGURE 11:**
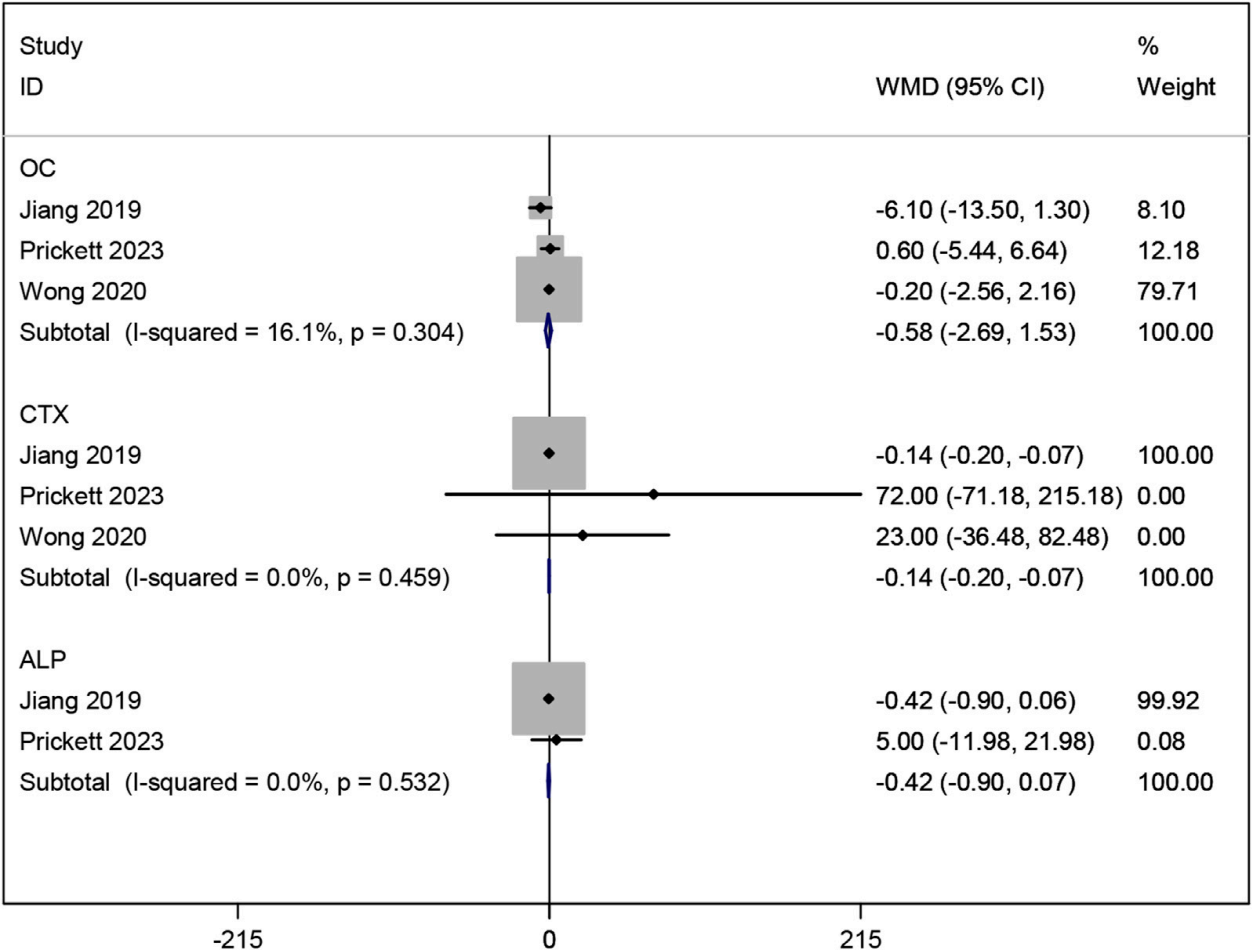
Forest plot for OC, CTX and ALP.

Three studies (Prickett et al., 2023; Wong et al., 2020; Jiang et al., 2019) investigated the effects of resveratrol on bone metabolic markers OC and CTX. Meta-analysis results showed no significant effect on OC (WMD: −0.581, 95% CI: −2.688 to 1.526, p = 0.589; Figure 11), but a significant improvement in CTX (WMD: −0.137, 95% CI: −0.204 to −0.070, p < 0.001; Figure 11). Two studies (Prickett et al., 2023; Jiang et al., 2019) examined ALP, showing no significant improvement with resveratrol compared to placebo (WMD: −0.416, 95% CI: −0.899 to 0.067, p = 0.092; Figure 11).

### 3.4 Sensitivity analysis

Heterogeneity was noted among the studies reporting RAVLT delayed *(*I^2^ = 62.8%; p = 0.068), Diastolic BP (*I* ^2^ = 56.1%; p = 0.103), pain VAS scores (*I* ^2^ = 57.7%; p = 0.124), and somatic menopausal symptoms (*I* ^2^ = 64.7%; p = 0.092), so sensitivity analysis were carried out to explore the potential causes. After excluding each study successively, the pooled WMD for the remaining RCTs did not change significantly, indicating that the result data are robust (Supplementary File S2).

### 3.5 Publication bias

To better assess the results of our study, despite there being fewer than 10 articles included in this study, the funnel plot was employed to evaluate publication bias. Visual assessment of the funnel plot and Egger’s test (P = 0.301) both suggested that there was no significant publication bias (Supplementary File S3).

## 4 Discussion

To our knowledge, this is the first meta-analysis summarizing and reporting the latest evidence on the effects of resveratrol on cognition and memory (processing speed, language, episodic memory, verbal memory, working memory, and cognitive flexibility), mood (depression and overall mood), metabolism (glucose, insulin, HOMA-IR, triglycerides, total cholesterol, LDL-cholesterol, HDL-cholesterol, systolic and diastolic BP), pain (pain scores, pain VAS scores, and PPI scores), sleep disturbance, menopausal symptoms (psychological, somatic, and urogenital menopausal symptoms), quality of life (SF-36 quality-of-life measure), and bone metabolic markers (CTX, ALP, and OC) in postmenopausal women. Two previous meta-analyses explored the therapeutic effects of resveratrol on bone health (Asis et al., 2019; Li et al., 2021). However, our study presents several key differences and innovations. Li’s study (Li et al., 2021) focused on the effects of resveratrol on bone quality in adults over 18 years old, with outcome measures including areal bone mineral density of total body, femoral neck, lumbar spine (L1-L4 or L2-L4), whole hip by dual-energy X-ray absorptiometry, ALP, bone alkaline phosphatase (BAP), parathyroid hormone (PTH), OC, CTX, N-terminal telopeptide of type I collagen, and procollagen I N-terminal propeptide (PINP). Asis’s study (Asis et al., 2019) investigated the effects of resveratrol on bone biomarkers in the general population, including ALP, BAP, Ca, PTH, CTX, PINP, and OC. In contrast, our study focuses exclusively on postmenopausal women, offering greater precision and relevance to this specific population. Moreover, our research provides a comprehensive assessment of the effects of resveratrol on postmenopausal women, not only in terms of bone health but also across multiple domains, including cognition and memory, mood, metabolism, pain, sleep disturbance, menopausal symptoms, and quality of life.

A total of 10 RCTs involving 928 participants were incorporated in our study. The meta-analysis results demonstrated significant improvements in pain score, pain VAS scales, PPI scales, and bone metabolic marker CTX following resveratrol treatment. However, interestingly, no significant changes were observed in the cognition and memory, mood, metabolism, sleep disturbance, menopausal symptoms, quality of life, and bone metabolic markers OC and ALP. Given the limited number of studies and participants, as well as clinical heterogeneity among the trials (e.g., varying dosages and treatment durations), our findings should be interpreted with caution.

Pain scores, pain VAS scores, and PPI scores are widely used tools for clinical pain assessment. Our meta-analysis results indicate that resveratrol has some beneficial effects on them, suggesting that it may potentially possess analgesic properties. While our meta-analysis identified statistically significant changes in pain scores, such as VAS and PPI scores, the effect sizes were small (e.g., WMD = −7.585 for pain VAS, p = 0.005). It is important to consider the clinical relevance of these changes. A typical minimal clinically important difference (MCID) for pain scores varies, but generally, the MCID typically ranges from 10% to 20% reduction on the Visual Analog Scale (VAS), depending on the population and pain type (Myles et al., 2017; Wickström and Edelstam, 2017). In our study, the observed reductions in pain scores, while statistically significant, may not meet the threshold for substantial clinical benefit. Therefore, while resveratrol may offer some relief, its practical implications in terms of improving patients’ day-to-day pain experiences may be modest. We recommend that future studies focus on larger sample sizes and longer treatment durations to better assess the clinical significance of resveratrol’s effects.

Both clinical and preclinical studies consistently report that resveratrol alleviates various types of pathological pain, including visceral pain (Lu et al., 2019), trigeminal neuralgia (Yin et al., 2019), diabetic neuropathy (Osmanlıoğlu and Nazıroğlu, 2024) and sciatica (Xu et al., 2018). Recently, Wang et al. systematically synthesized these findings, revealing that the analgesic mechanisms of resveratrol involve the regulation of key signaling pathways, such as TNFR1/NF-κB, PI3K/Akt/mTOR, Nrf2, Sirt1, and MAPK (Wang et al., 2024). In the two studies included in this study, the primary pain reported by postmenopausal women was chronic musculoskeletal pain, commonly associated with age-related osteoarthritis (Thaung Zaw et al., 2020a; Wong et al., 2017). The decrease in estrogen levels after menopause alters the physiological functions of muscles, tendons, ligaments, and bones, reducing their resistance to mechanical stress and increasing susceptibility to pain (Hansen, 2018; Strand et al., 2024). Additionally, joint structural damage and the loss of blood supply and nutrients to previously perfused tissues can lead to ischemic pain. Other studies suggest that pro-inflammatory mediators, such as TNF-α, IL-6, and IL-8, enter the circulation and create a systemic inflammatory environment, which may lead to endothelial dysfunction and exacerbate pain (Jiang et al., 2022; Wei et al., 2020). Resveratrol can increase the bioavailability of nitric oxide, promoting vasodilation and improving blood flow in affected tissues, thereby alleviating pain (Thaung Zaw et al., 2020a). *In vitro* study has shown that resveratrol reduces IL-1β levels in human primary chondrocytes by downregulating the NF-κB pathway (Yang et al., 2022). Furthermore, in a short-term study on patients with mild to moderate knee osteoarthritis, Marouf et al. found that resveratrol combined with meloxicam reduced pain severity and inflammatory markers (TNF-α, IL-1β, IL-6, and C-reactive protein) compared to placebo and meloxicam alone (Marouf et al., 2018). Overall, the analgesic effects of resveratrol are attributed to its anti-inflammatory and antioxidant properties, suggesting its potential as a therapeutic option for chronic pain management in postmenopausal women. However, given the limited number of studies in our research, larger and more comprehensive studies should be conducted in the future to confirm its analgesic effects and explore the underlying mechanisms.

Analysis of bone metabolic markers suggests that resveratrol can lower CTX levels, indicating it may have some potential protective effects on bone health. However, no significant impact was observed on OC and ALP. Current studies on the effects of resveratrol on bone metabolism have yielded inconsistent results. A meta-analysis by Asis found that resveratrol reduced ALP levels but had no significant effects on CTX and OC (Asis et al., 2019), while Li’s meta-analysis indicated that resveratrol had no effect on OC, ALP, and CTX (Li et al., 2021). These conflicting results suggest that the impact of resveratrol on bone metabolism remains inconclusive, and further large-scale clinical research is necessary to clarify this issue.

While our meta-analysis did not demonstrate significant improvements in cognition, memory, mood, sleep disturbance, menopausal symptoms, or quality of life for postmenopausal women—contrary to previous reports of benefits in elderly populations (Fekete et al., 2023; Tu et al., 2023; Witte et al., 2014)—this apparent discrepancy necessitates a multifaceted exploration. Mechanistically, resveratrol modulates critical neuroprotective pathways including SIRT1-mediated deacetylation of PGC-1α (enhancing mitochondrial biogenesis in hippocampal neurons), suppression of NF-κB-driven neuroinflammation in prefrontal regions, and BDNF upregulation via CREB phosphorylation to promote synaptic plasticity (Azargoonjahromi et al., 2025; Barbalho et al., 2025; Shaito et al., 2023). However, menopause is a dynamic neuroendocrine transition marked by estrogen depletion and associated with unique neuropathological changes. This hormonal shift significantly impacts brain structure, connectivity, and metabolic profiles during endocrine aging. Menopause accelerates β-amyloid (Aβ) deposition within the limbic system, a process further exacerbated in postmenopausal women carrying the apolipoprotein E-4 (APOE-4) genotype. This enhanced Aβ accumulation may trigger microglial overactivation and lead to the loss of cortical GABAergic interneurons, thereby creating a neurobiological environment in which standard resveratrol supplementation may be insufficient (Liang et al., 2024; Mosconi et al., 2021; Wang et al., 2025). Methodologically, three interrelated limitations may account for the null findings observed in our study. First, the variability in resveratrol dosing among included trials may have resulted in suboptimal central nervous system bioavailability, as indicated by low brain-to-plasma concentration ratios. Second, the relatively short intervention durations may have been inadequate to detect structural neuroplastic changes. Third, the use of heterogeneous cognitive assessment tools—ranging from the Montreal Cognitive Assessment (MoCA) to various verbal memory tests—may lack sensitivity to the specific cognitive domains most affected during the menopausal transition. Moreover, factors such as social support, psychological status, and lifestyle can influence mood, metabolism, sleep disturbance, menopausal symptoms, and quality of life, suggesting that resveratrol alone may not yield substantial improvements in these domains. Therefore, future research should explore the potential of combination therapies. Recent studies also suggest that lower doses of resveratrol may produce more significant effects, providing new avenues for preclinical research and indicating the need for further exploration of its pharmacological effects to determine optimal doses for clinical trials (Samuel et al., 2019; Vikal et al., 2024). However, the low blood concentration of resveratrol, along with its rapid absorption and clearance, complicates the accurate assessment of its effective dose (Robertson et al., 2022; Salla et al., 2024). Therefore, future efforts should focus on developing resveratrol delivery systems with higher bioavailability and analogs to optimize clinical effectiveness. Furthermore, due to the lack of stringent regulations, dietary supplement manufacturers face fewer requirements for proving product efficacy, safety, and quality compared to the pharmaceutical industry. This has led to many products being potentially ineffective, with significant variability in quality among different manufacturers. Stricter regulations should be implemented in the nutritional supplement industry, as many promising therapeutic options may emerge from this field.

Our study has several strengths. First, it is the first systematic review and meta-analysis investigating the effects of resveratrol on postmenopausal women. Second, to comprehensively assess the impact of resveratrol, a wide range of subjective and objective outcome measures were employed, including cognitive and mood assessments, pain scores, metabolic markers, and bone health indicators. These measures are summarized in Table 3 to provide a more organized presentation of the findings. Moreover, most of the included studies were of high quality, and the heterogeneity of the combined data was low. Finally, sensitivity analysis indicated that the meta-analysis results are relatively robust, and funnel plots and Egger’s test revealed no evidence of publication bias.

**TABLE 3:** Outcome measures assessed in the study.

Category	Outcome measure	Description
Subjective indicators	PCT (Pattern Comparison Speed Test)	Cognitive processing speed
	TMT A (Trail Making Task A)	Cognitive flexibility
	PVT (Picture Vocabulary Test)	Verbal memory
	ORR (Oral Reading Recognition Test)	Reading ability
	PSM (Picture Sequence Memory Test)	Episodic memory
	RAVLT Immediate and Delayed (Rey Auditory Verbal Learning Test)	Verbal memory (immediate and delayed recall)
	LSWM (List Sorting Working Memory Test)	Working memory
	FSS (Forward Spatial Span Test)	Spatial memory
	DCCS (Dimensional Change Card Sort Test)	Cognitive flexibility
	FICA (Flanker Inhibitory Control and Attention Test)	Attention and inhibitory control
	TMT Performance (Trail Making Task performance)	Cognitive performance (general)
	Depression (CES-D: Centre for Epidemiologic Studies Depression Scale)	Mood
	Overall Mood	Self-reported mood
	Pain scores (VAS, PPI)	Pain intensity and pain interference
	Sleep disturbance	Sleep quality and disturbances
	Psychological, somatic, and urogenital menopausal symptoms	Menopausal symptom severity
	SF-36 Quality of Life	Quality of life (general health and wellbeing)
Objective indicators	Glucose, Insulin, HOMA-IR (Homeostasis Model Assessment of Insulin Resistance)	Metabolic health markers
	Triglycerides, Total Cholesterol, LDL/HDL Cholesterol	Lipid profile (blood fat levels)
	Systolic BP, Diastolic BP	Blood pressure
	CTX (C-terminal telopeptide of type 1 collagen)	Bone resorption marker
	ALP (Alkaline Phosphatase), OC (Osteocalcin)	Bone metabolism markers

However, some limitations must be considered. Firstly, certain methodological limitations were present in individual studies. The risk of bias assessment indicated that most studies had a low risk of bias across several domains. However, the variation in study quality, especially regarding blinding and random sequence generation, may have influenced the findings. Specifically, three out of the ten included studies did not provide sufficient information regarding the randomization process, one study did not report allocation concealment, and one study did not mention double-blinding. Studies with higher quality ratings (e.g., Prickett et al., 2023; Thaung Zaw et al., 2020a) showed more robust outcomes in pain management and bone metabolic markers. Nonetheless, studies with unclear or higher risk in randomization and blinding (e.g., Evans et al., 2017; Jiang et al., 2019) demonstrated more variable results, particularly in cognition and mood. Therefore, while resveratrol’s effects on pain and bone metabolism appear promising, caution is warranted in interpreting its efficacy in other areas due to the potential influence of study quality. Secondly, heterogeneity was observed in studies examining the relationship between resveratrol and RAVLT delayed, Diastolic BP, pain VAS scores and somatic menopausal symptoms. However, due to the limited number of studies, subgroup analysis and meta-regression could not be conducted to assess the impact of variables such as dose, intervention duration, and region. Nevertheless, we performed a qualitative comparison to explore potential sources of heterogeneity. The heterogeneity in RAVLT delayed could mainly arise from clinical and methodological differences: (1) Sample size: The study by Evans et al. (2017) had a sample size of 79, while Thaung Zaw et al. (2020a) and Thaung Zaw et al. (2021) had larger sample sizes of 125; (2) Age: The average age in Evans et al. (2017) was 61.5 years, lower than in the other two studies, which averaged 65 years; (3) Treatment duration: Evans et al. (2017) had a treatment duration of 14 weeks, much shorter than Thaung Zaw et al. (2020a) (12 months) and Thaung Zaw et al. (2021) (24 months); (4) Methodology: Evans et al. (2017) did not provide detailed information on the randomization process, which may have introduced selection bias. The heterogeneity in diastolic blood pressure could be attributed to: (1) Sample size: The studies by Ozemek et al. (2020) and Yoshino et al. (2012) had sample sizes of 15 and 29, respectively, while Thaung Zaw et al. (2021) had a larger sample size of 125; (2) Age: The average age in Thaung Zaw et al. (2021) was higher compared to Ozemek et al. (2020) and Yoshino et al. (2012); (3) Treatment duration: Ozemek et al. (2020) did not report treatment duration, while Thaung Zaw et al. (2021) had a significantly longer duration of 24 months compared to Yoshino et al. (2012) (12 weeks); (4) Drug dosage: Ozemek et al. (2020) used a higher dose (250 mg/d) compared to the other two studies (150 mg/d); (5) Region: Thaung Zaw et al. (2021) was conducted in Australia, while the other two studies were in the America; (6) Methodology: Except for the study by Thaung Zaw et al. (2021), the other two studies did not provide detailed descriptions of the randomization methods. The heterogeneity in pain VAS scores and somatic menopausal symptoms could be attributed to: (1) Sample size: Thaung Zaw et al. (2020b) had a sample size of 146, while Wong et al. (2017) had 72; (2) Treatment duration: Thaung Zaw et al. (2020b) had a treatment duration of 12 months, while Wong et al. (2017) had 14 weeks. Despite these sources of heterogeneity, the effect directions remained consistent across all studies, and sensitivity analyses confirmed the robustness of the results. Furthermore, the small sample sizes in some studies may have resulted in insufficient statistical power. Therefore, while resveratrol supplementation demonstrated statistically significant effects on pain and bone metabolism markers, the clinical relevance of these findings remains uncertain due to the small effect sizes. This is particularly evident in pain relief, where the observed changes may not result in substantial improvements in daily functioning. Future research should aim to better quantify the clinical significance of resveratrol’s effects, including the use of clinically meaningful outcome measures and larger, longer-term trials. In particular, in-depth studies on the mechanisms by which resveratrol improves neurovascular coupling and pain in postmenopausal women are essential for a better understanding of its potential benefits and applications.

## 5 Conclusion

This systematic review and meta-analysis indicates that resveratrol supplementation may play a role in improving pain and CTX levels in postmenopausal women. However, its effects on cognition and memory, mood, metabolism, sleep disturbance, menopausal symptoms, quality of life, OC, and ALP remain uncertain. These findings imply that although resveratrol provides potential benefits in specific health domains, its overall efficacy may be restricted or conditional. Giving the limited number of available RCTs and observed heterogeneity, future large-scale, multicenter clinical trials with long-term follow-up are requisite to further explore resveratrol’s health effects in postmenopausal women and determine optimal dosages, treatment durations, and combinations with other interventions.

## Data Availability

The original contributions presented in the study are included in the article/Supplementary Material, further inquiries can be directed to the corresponding authors.
