# Supplementary material for: Effects of resveratrol on postmenopausal women: a systematic review and meta-analysis
Source: Front Pharmacol. 2025 Jul 23;16:1588284. doi: 10.3389/fphar.2025.1588284 (PMC12325339; doi:10.3389/fphar.2025.1588284)
Supplement: Supplementary file 1 [file DataSheet3.doc]

Supplementary Appendix 3

**publication bias**

1. **Funnel plot**

**(B) Egger’s test**
