# Supplementary material for: Effects of resveratrol on postmenopausal women: a systematic review and meta-analysis
Source: Front Pharmacol. 2025 Jul 23;16:1588284. doi: 10.3389/fphar.2025.1588284 (PMC12325339; doi:10.3389/fphar.2025.1588284)
Supplement: Supplementary file 2 [file DataSheet2.docx]

Supplementary Appendix 2

**Sensitivity analysis**

(1)RAVLT delayed

(2)Diastolic BP

(3)VAS

(4)Somatic menopausal symptoms
