# Supplementary material for: Effects of resveratrol on postmenopausal women: a systematic review and meta-analysis
Source: Front Pharmacol. 2025 Jul 23;16:1588284. doi: 10.3389/fphar.2025.1588284 (PMC12325339; doi:10.3389/fphar.2025.1588284)
Supplement: Supplementary file 3 [file DataSheet1.docx]

Supplementary Appendix 1

**Details of Search Strategy**

**Source: PubMed; Searched on: January 2025;**

**Results: 87**

| **Search** | **Query** |
| --- | --- |
| #1 | “Resveratrol”[Mesh] |
| #2 | Resveratrol[Title/Abstract] |
| #3 | Trans-resveratrol[Title/Abstract] |
| #4 | #1 OR #2 OR #3 |
| #5 | Postmenopausal women[Title/Abstract] |
| #6 | Senile women[Title/Abstract] |
| #7 | Female[Title/Abstract] |
| #8 | Women[Title/Abstract] |
| #9 | #4 OR #5 OR #6 OR #7 OR #8 |
| #10 | Random controlled trial[Title/Abstract] |
| #11 | Random[Title/Abstract] |
| #12 | Randomized[Title/Abstract] |
| #13 | Controlled[Title/Abstract] |
| #14 | RCT[Title/Abstract] |
| #15 | #10 OR #11 OR #12 OR #13 OR #14 |
| #16 | #4 AND #9 AND #15 |

**Source: Cochrane Library; Searched on: January 2025; Results: 354**

| **Search** | **Query** |
| --- | --- |
| #1 | MeSH descriptor: [Resveratrol] explode all trees |
| #2 | (Resveratrol):ti,ab,kw |
| #3 | (trans-resveratrol):ti,ab,kw |
| #4 | #1 OR #2 OR #3 |
| #5 | (Postmenopausal women):ti,ab,kw |
| #6 | (Senile women):ti,ab,kw |
| #7 | (Female):ti,ab,kw |
| #8 | (Women):ti,ab,kw |
| #9 | #4 OR #5 OR #6 OR #7 OR #8 |
| #10 | (Random controlled trial):ti,ab,kw |
| #11 | (Random):ti,ab,kw |
| #12 | (Randomized):ti,ab,kw |
| #13 | (Controlled):ti,ab,kw |
| #14 | (RCT):ti,ab,kw |
| #15 | #10 OR #11 OR #12 OR #13 OR #14 |
| #16 | #4 AND #9 AND #15 |

**Source: Embase; Searched on: January 2025; Results: 161**

| **Search** | **Query** |
| --- | --- |
| #1 | 'Resveratrol'/exp |
| #2 | 'Resveratrol':ab,ti |
| #3 | 'trans-resveratrol':ab,ti |
| #4 | #1 OR #2 OR #3 |
| #5 | 'Postmenopausal women':ab,ti |
| #6 | 'Senile women':ab,ti |
| #7 | 'Female':ab,ti |
| #8 | 'Women':ab,ti |
| #9 | #4 OR #5 OR #6 OR #7 OR #8 |
| #10 | 'Random controlled trial':ab,ti |
| #11 | 'Random':ab,ti |
| #12 | 'Randomized':ab,ti |
| #13 | 'RCT':ab,ti |
| #14 | 'Controlled':ab,ti |
| #15 | #10 OR #11 OR #12 OR #13 OR #14 |
| #16 | #4 AND #9 AND #15 |

**Source: Web of Science; Searched on: January 2025; Results: 6**

| **Search** | **Query** |
| --- | --- |
| #1 | TS="Resveratrol" |
| #2 | TS="trans-resveratrol" |
| #3 | #1 OR #2 |
| #4 | TS="Postmenopausal women " |
| #5 | TS="Senile women" |
| #6 | TS="women" |
| #7 | TS="Female" |
| #8 | #4 OR #5 OR #6 OR #7 |
| #9 | TS="Random controlled trial" |
| #10 | TS="Random" |
| #11 | TS="Randomized" |
| #12 | TS="Controlled" |
| #13 | TS="RCT" |
| #14 | #9 OR #10 OR #11 OR #12 OR #13 |
| #15 | #3 AND #8 AND #14 |

**Search in Chinese: CNKI; Searched on: January 2025; Results: 64**

TKA=(liluchun+fanshililuchu) AND TKA=(juejinghoufunv+juejinghounvxing+laoniannvxing+nv) AND FT=(suiji)

**Search in Chinese: Wanfang Database; Searched on: January 2025; Results: 100**

Zhuti:(liluchun+fanshililuchu) * Zhuti:(juejinghoufunv+juejinghounvxing+laoniannvxing+nv)*Quanbu:(suiji)

**Search in Chinese: VIP database; Searched on: January 2025; Results: 7**

(M=liluchun+fanshililuchu)*(M=juejinghoufunv+juejinghounvxing+laoniannvxing+nv)*(M=suiji)
